# Improved calibration of electrochemical aptamer-based sensors

**DOI:** 10.1038/s41598-022-09070-7

**Published:** 2022-04-01

**Authors:** Alex M. Downs, Julian Gerson, Kaylyn K. Leung, Kevin M. Honeywell, Tod Kippin, Kevin W. Plaxco

**Affiliations:** 1 Department of Mechanical Engineering, University of California Santa Barbara, Santa Barbara, CA 93106 USA; 2 Center for Bioengineering, University of California Santa Barbara, Santa Barbara, CA 93106 USA; 3 Department of Psychological and Brain Sciences, University of California Santa Barbara, Santa Barbara, CA 93106 USA; 4 The Neuroscience Research Institute and Department of Molecular Cellular and Developmental Biology, University of California Santa Barbara, Santa Barbara, CA 93106 USA; 5 Department of Chemistry and Biochemistry, University of California Santa Barbara, Santa Barbara, CA 93106 USA

**Keywords:** DNA probes, Sensors, Bioanalytical chemistry, Electrochemistry, Biomedical engineering, Biomarkers, Health care, Chemistry, Engineering

## Abstract

Electrochemical aptamer-based (EAB) sensors support the real-time, high frequency measurement of pharmaceuticals and metabolites in-situ in the living body, rendering them a potentially powerful technology for both research and clinical applications. Here we explore quantification using EAB sensors, examining the impact of media selection and temperature on measurement performance. Using freshly-collected, undiluted whole blood at body temperature as both our calibration and measurement conditions, we demonstrate accuracy of better than ± 10% for the measurement of our test bed drug, vancomycin. Comparing titrations collected at room and body temperature, we find that matching the temperature of calibration curve collection to the temperature used during measurements improves quantification by reducing differences in sensor gain and binding curve midpoint. We likewise find that, because blood age impacts the sensor response, calibrating in freshly collected blood can improve quantification. Finally, we demonstrate the use of non-blood proxy media to achieve calibration without the need to collect fresh whole blood.

## Introduction

**Figure 1:**
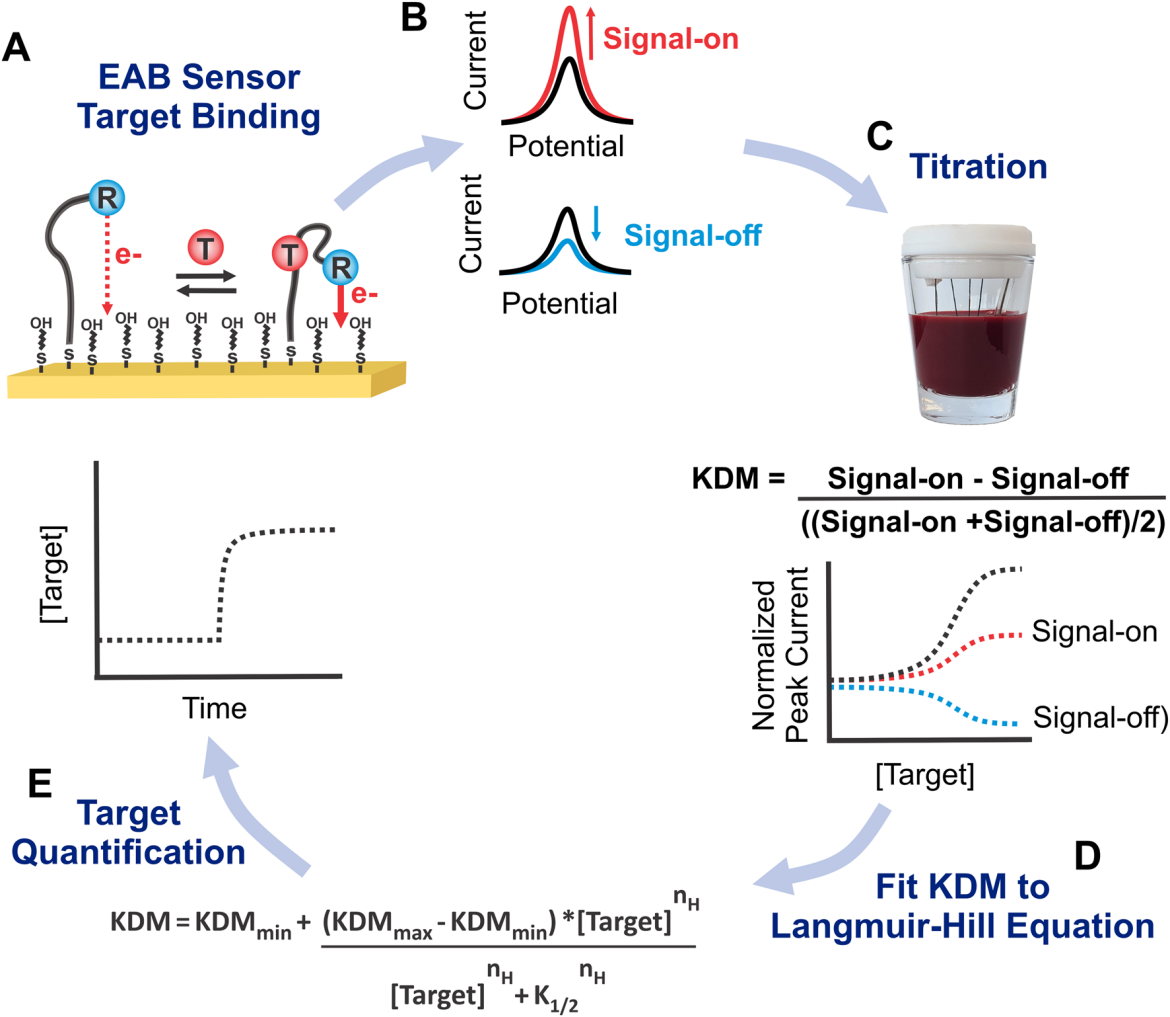
(**A**) EAB sensors consist of a redox-reporter-modified (R) aptamer attached to a gold electrode coated with a passivating self-assembled monolayer (here 6-mercapto-1-hexanol). Upon binding of target (T), a change in electron transfer kinetics between the redox reporter and the surface occurs, which is easily monitored using square wave voltammetry. (**B**) Depending upon the square-wave frequency employed, the peak currents seen in square wave voltammetry will increase (“signal on” behavior) or decrease (“signal off”) in response to target binding. (**C**) To determine responses at different frequencies, we titrate the sensors with known amounts of target. To increase gain and correct for the appearance of drift, we obtain Kinetic Differential Measurement (KDM) values by taking the difference in normalized peak currents collected at a signal-on and a signal-off frequency, then dividing that value by the average of the signal-on and signal-off peak currents. (**D**) We then fit the KDM values to a Hill-Langmuir equation, extracting parameters for KDM_max_ (the maximum KDM response, or gain) KDM_min_ and K_1/2_. (**E**) Using these parameters, we convert observed KDM measurements into concentration estimates.

Electrochemical aptamer-based (EAB) sensors are a specific class of aptasensors that, uniquely, support high-frequency^1,2^ real-time molecular measurements^3,4^ directly in complex biological media, including unprocessed, undiluted bodily fluids^5,6^. In this sensor architecture, a target-recognizing aptamer is modified with a redox reporter and covalently attached to a gold electrode using a self-assembled monolayer (Fig. 1A)^5,9^. Upon target binding, the aptamer undergoes a conformational change, producing an easily-measurable shift in electrochemical signal^1^. As these sensors include both a recognition element (the aptamer) and a signaling element (the redox reporter), they do not require washing steps or reagent addition. And because they maintain their signaling properties in even complex sample matrices, such as undiluted whole blood, they perform well even when placed in situ in the living body^2–7^. Indeed, their ability to perform real-time molecular measurements in vivo even supports closed-loop feedback-controlled drug delivery^8,9^. Their utility for in-vivo measurements makes this class of sensors particularly promising for clinical health monitoring and wearable devices.

While a number of electrochemical techniques^5,10–12^ have been used to interrogate EAB sensors, converting the resulting signals into estimated target concentrations always relies on the use of a calibration curve. When applying square wave voltammetry, the most commonly employed interrogation technique, a calibration curve is produced by collecting voltammogram peak currents over a range of target concentrations (Fig. 1C). Of note, because EAB sensor signaling varies with applied square wave frequency, square wave voltammetry can be tuned to yield an increase (“signal-on”) or a decrease (“signal-off”) in peak current upon target addition (Fig. 1B)^1,13–15^. To correct for drift and enhance gain during in vivo measurements, voltammograms are collected at two such frequencies and converted into “Kinetic Differential Measurement” (KDM) values. These KDM values are derived by subtracting the normalized peak currents seen at signal-on and signal-off frequencies, then dividing by their average (Fig. 1C)^16^. To generate a calibration curve, the averaged KDM values collected in vitro over a range of target concentrations are fitted to a Hill-Langmuir isotherm [Eq. 1, Fig. 1D]^17^:

1 $$\mathrm{KDM}={\mathrm{KDM}}_{\mathrm{min}}+ \frac{\left({\mathrm{KDM}}_{\mathrm{max }}- {\mathrm{KDM}}_{\mathrm{min}}\right)*{[\mathrm{Target}]}^{{\mathrm{n}}_{\mathrm{H}}}}{{[\mathrm{Target}]}^{{\mathrm{n}}_{\mathrm{H}}}+ {\mathrm{K}}_{1/2}^{{\mathrm{n}}_{\mathrm{H}}}}$$

where n_H_ is the Hill coefficient (a measure of binding cooperativity), K_1/2_ is the midpoint of the binding curve, KDM is the KDM value observed at the applied target concentration, KDM_min_ is the KDM value seen in the absence of target, and KDM_max_ is the KDM value expected at saturating target. These parameters extracted from a calibration curve, then, allow translation of EAB sensor output into estimates of target concentration [Eq. 2, Fig. 1E]:

2 $$\left[\mathrm{Target}\right]={\sqrt[{\mathrm{n}}_{\mathrm{H}}]{\frac{{\mathrm{K}}_{1/2}^{{\mathrm{n}}_{\mathrm{H}}}*(\mathrm{ KDM}- {\mathrm{KDM}}_{\mathrm{min}})}{{\mathrm{KDM}}_{\mathrm{max }}-\mathrm{ KDM}}}}$$

EAB calibration curves thus depend on both the affinity of the aptamer, K_1/2_ (which, in the case of a non-cooperative aptamer, simplifies to K_D_, the dissociation constant), the cooperativity (or anti-cooperativity) of binding, n_H_, and the sensor’s signal gain, KDM_max_. Given that both of these parameters are influenced by environmental factors, such as temperature^18,19^, pH^20^, and ionic strength^20,21^, careful selection of calibration conditions is required in order to accurate and precise measurements. Here, we examine the calibration of EAB sensors in detail, with the aim of improving the accuracy of in vivo measurements performed using them. Specifically, we investigate the impact of media temperature, age, and composition on the observed calibration curves obtained using square wave voltammetry, and assess the accuracy of in vitro measurements performed in freshly-collected, body temperature blood. As our test bed, we examine the response for a sensor detecting vancomycin, an antibiotic target^22^ of strong interest for therapeutic monitoring^23–25^.

## Results and discussion

**Figure 2:**
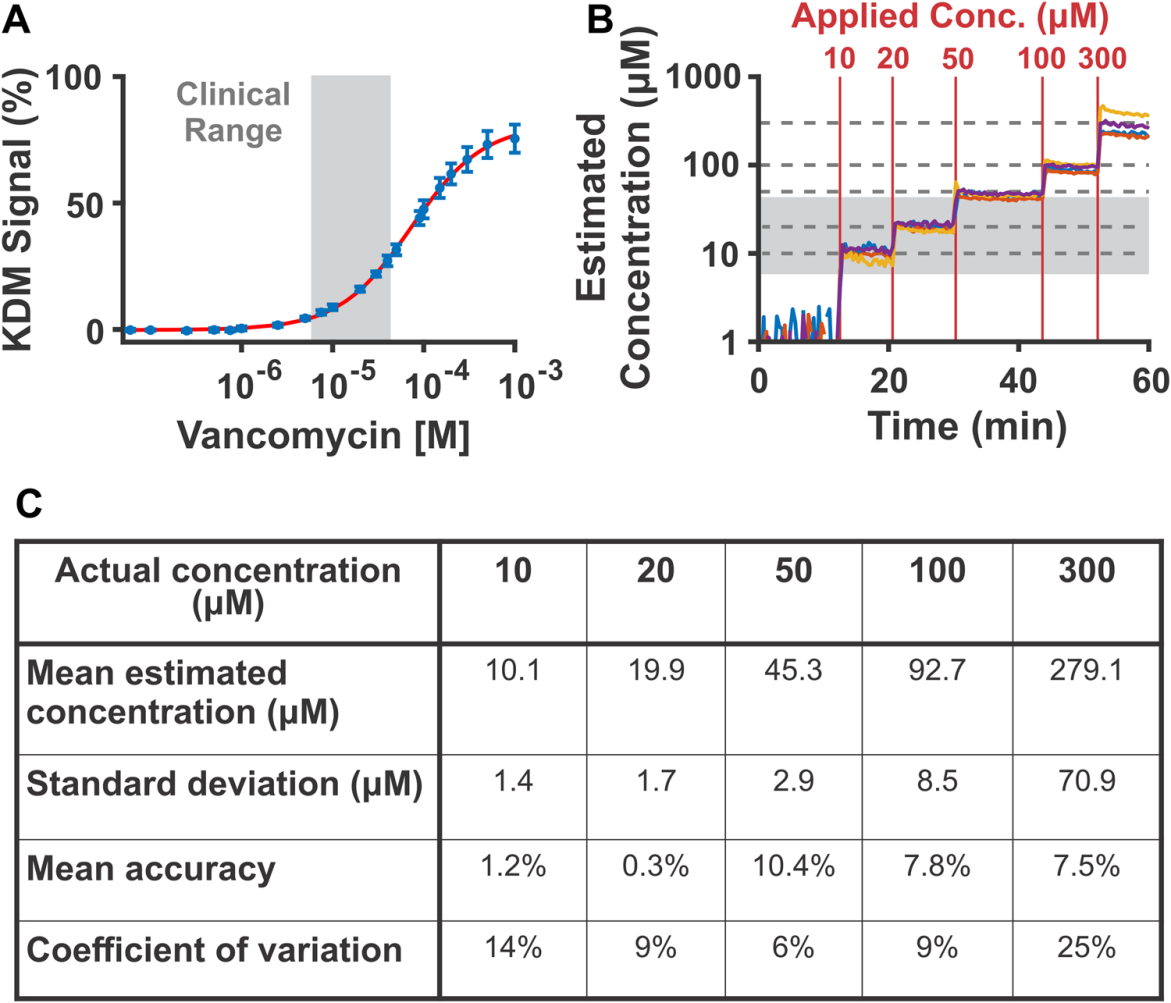
Vancomycin-detecting EAB sensors calibrated using a calibration curve collected in matched media and temperature easily achieve clinically useful measurement accuracy. (**A**) Here we created a calibration curve by fitting the average response of four sensors to a Hill-Langmuir isotherm using data collected at 37 °C in freshly-collected rat blood. We derive KDM values by subtracting the normalized peak heights collected at 300 Hz from those collected at 25 Hz, and dividing by their average^16^. We indicate the clinical range of vancomycin in grey. Specifically, the clinical window of vancomycin ranges from 6 to 42 µM, which reflects the minimum target concentrations to achieve clinical effect, to the mean maximum peak concentrations concentrations^26,27^. The error bars shown here and in the following figures reflect the standard deviation of replicate measurements performed using independent sensors (K_1/2_ = 73 ± 4 µM, n = 4). The error reported for these and all other K_1/2_ values reflect 95% confidence intervals. (**B**) We then apply this calibration curve to quantify measurements performed using the same four sensors in 37 °C fresh rat blood to which 10, 20, 50, 100, or 300 µM vancomycin has been added (dotted lines, red lines). We indicate the clinical range of vancomycin in grey. (**C**) From these measurements, we calculate mean estimated concentration, standard deviation, mean accuracy (defined as 100*(expected – observed)/observed), and coefficient of variation (100*population standard deviation/population mean). We observe better than 10% accuracy for all challenges.

EAB sensors calibrated in fresh whole rat blood under the same conditions employed during the measurement achieve clinically useful performance. To demonstrate this, we applied parameters obtained from a calibration curve collected in fresh, body temperature (37 °C) rat blood (Fig. 2A) to measurements performed in fresh, body temperature rat blood dosed with known concentrations of vancomycin (Fig. 2B). Doing so, we achieve mean accuracy of 1.2% or better over the drug’s 6 to 42 µM clinically relevant range^26,27^ and 10.4% or better at all concentrations (Fig. 2C). Here, we define accuracy as the mean of the relative difference between the estimated and applied concentrations (100*(expected – observed)/observed). Likewise, we achieve precision of 14% or better in the clinical range, which we define as the coefficient of variation (100*population standard deviation/population mean). This level of performance is more than adequate for many clinical applications. For example, accuracy of ± 20% over the therapeutic range is sufficient to achieve clinically relevant therapeutic monitoring of vancomycin^24,28^. This suggests that for measurements in the living body, applying a calibration curve captured in freshly collected, body temperature blood will yield clinically accurate and precise measurements of this drug.

**Figure 3:**
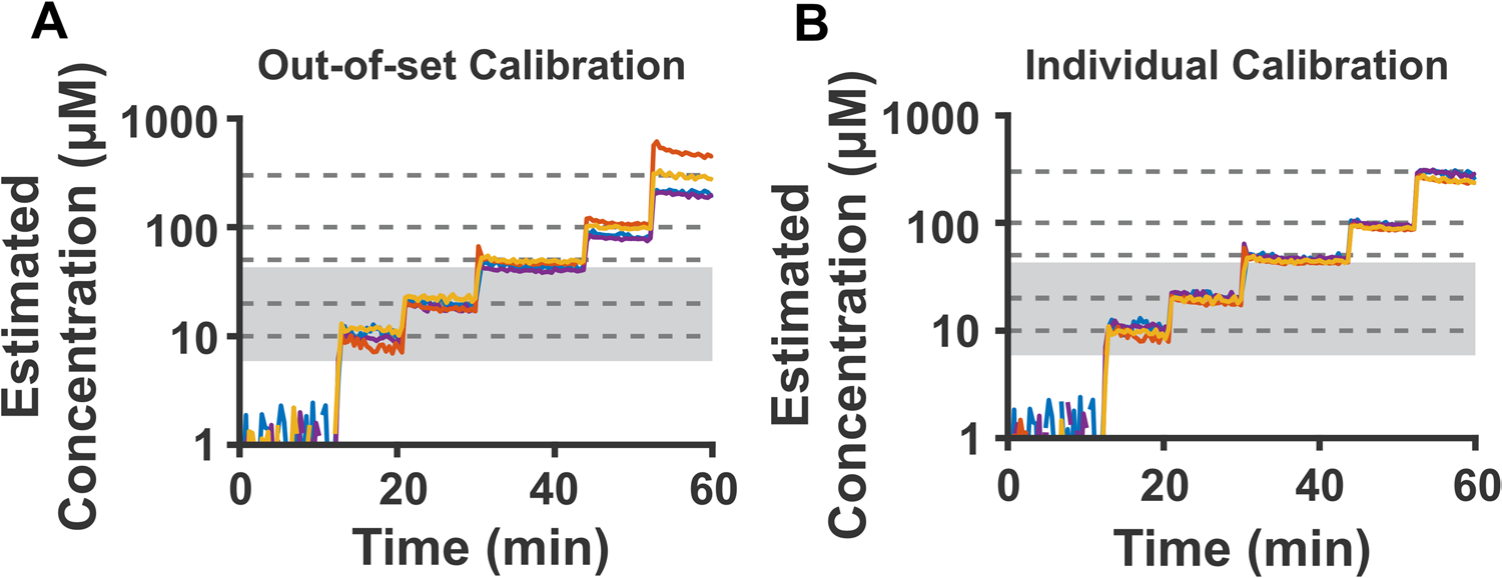
Calibrating sensors using out-of-set data, or calibrating individual sensors to their respective calibration curve does not greatly change sensor accuracy in vancomycin’s clinical range (highlighted in grey). (**A**) To calibrate sensors “out-of-set,” for each sensor, we calibrate using a calibration curve formed from the other three sensors. (**B**) To “individually” calibrate the sensors, we use the calibration curve collected for a specific sensor to quantify its own resulting dose–response curve.

In the studies described above, we calibrated each individual sensor using a single, common calibration curve obtained by averaging the sensor’s calibration curve with those of other vancomycin-detecting sensors. That is, each sensor was calibrated using a calibration curve to which it itself had also contributed. If, however, we instead use an “out-of-set” calibration curve (a calibration curve to which the sensor under investigation did not contribute, Fig. 3A), we see no significant change in accuracy and only a slight reduction in precision over the clinically relevant range of vancomycin concentrations (Table S1). Likewise, we do not see significant improvement in accuracy or precision over the clinical range if we calibrate each sensor using its own, individual calibration curve (Fig. 3B, Table S2). These observations suggest that sensor-to-sensor variation is not a major contributor to the level of accuracy and precision we achieve with this sensor at clinical target concentrations.

**Figure 4:**
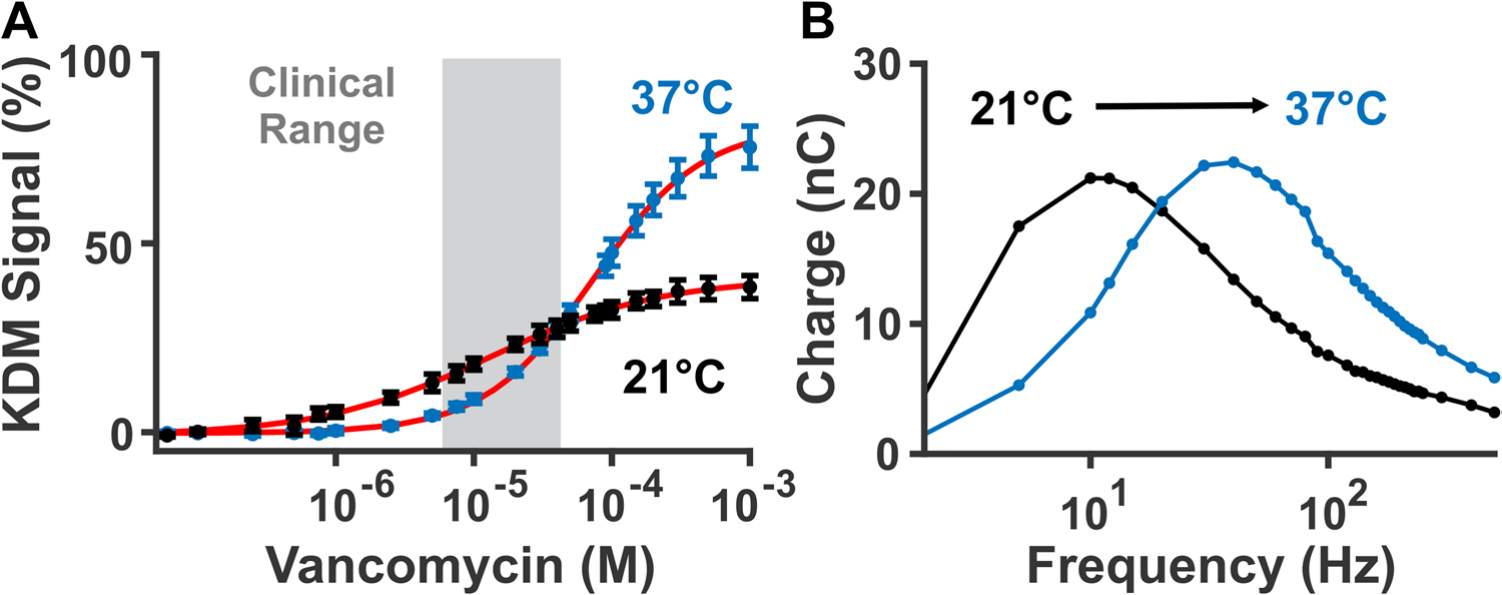
EAB signaling changes significantly between room and body temperature. (**A**) For example, calibration curves measured in freshly collected rat blood at 21 °C (black, n = 4 sensors, K_1/2_ = 13 ± 2 µM) and 37 °C (blue, n = 4 sensors, K_1/2_ = 73 ± 4 µM) differ in their binding midpoint, Hill coefficient, and signal gain (Table S3). (**B**) This occurs at least in part because the electron transfer rate from the redox reporter changes with temperature. Specifically, the peak charge transfer rate shifts toward higher frequencies at higher temperatures. To illustrate this, we plot here charge transfer versus frequency for a representative sensor in absence of target. We do so by determining square wave voltammogram peak current, and dividing it by its given interrogation frequency (additional sensor curves shown in Figure S1)^29^.

Collecting calibration curves at the appropriate temperature is key to sensor calibration. Specifically, between room and body temperature, calibration curves differ significantly (Fig. 4A). Depending on interrogation frequency employed, this can lead to considerable under- or over-estimation of target concentrations. Interrogating at 25 and 300 Hz, for example, we observe up to a 10% higher KDM signal at room temperature than at body temperature over vancomycin’s clinical concentration range. Consequently, applying a calibration curve collected at room temperature to those collected at body temperature causes substantial concentration underestimates (Figure S2). Interrogating using other square wave frequency pairs, likewise, can yield even more significant differences in KDM signal in the clinical range (Figure S3). These differences in sensor response occur because temperature changes can shift system properties, such as binding equilibrium coefficients and the electron transfer rate itself. For example, the electron transfer rate (indicated by the location of peak charge transfer, when plotting interrogation frequency versus charge transfer) increases with temperature for the vancomycin aptamer (Fig. 4B), as well as for EAB sensors against other targets (Figures S4–S6). This shift is great enough that it can affect the selection of signal-on and signal-off frequencies. For example, from room temperature to body temperature, 25 Hz changes from a weak signal-on frequency to a clear signal-off frequency (Figure S7). These results illustrate the importance of media temperature for both the accurate calibration of in vivo data, as well as the selection of the signal-on and signal-off frequencies used to perform KDM.

**Figure 5:**
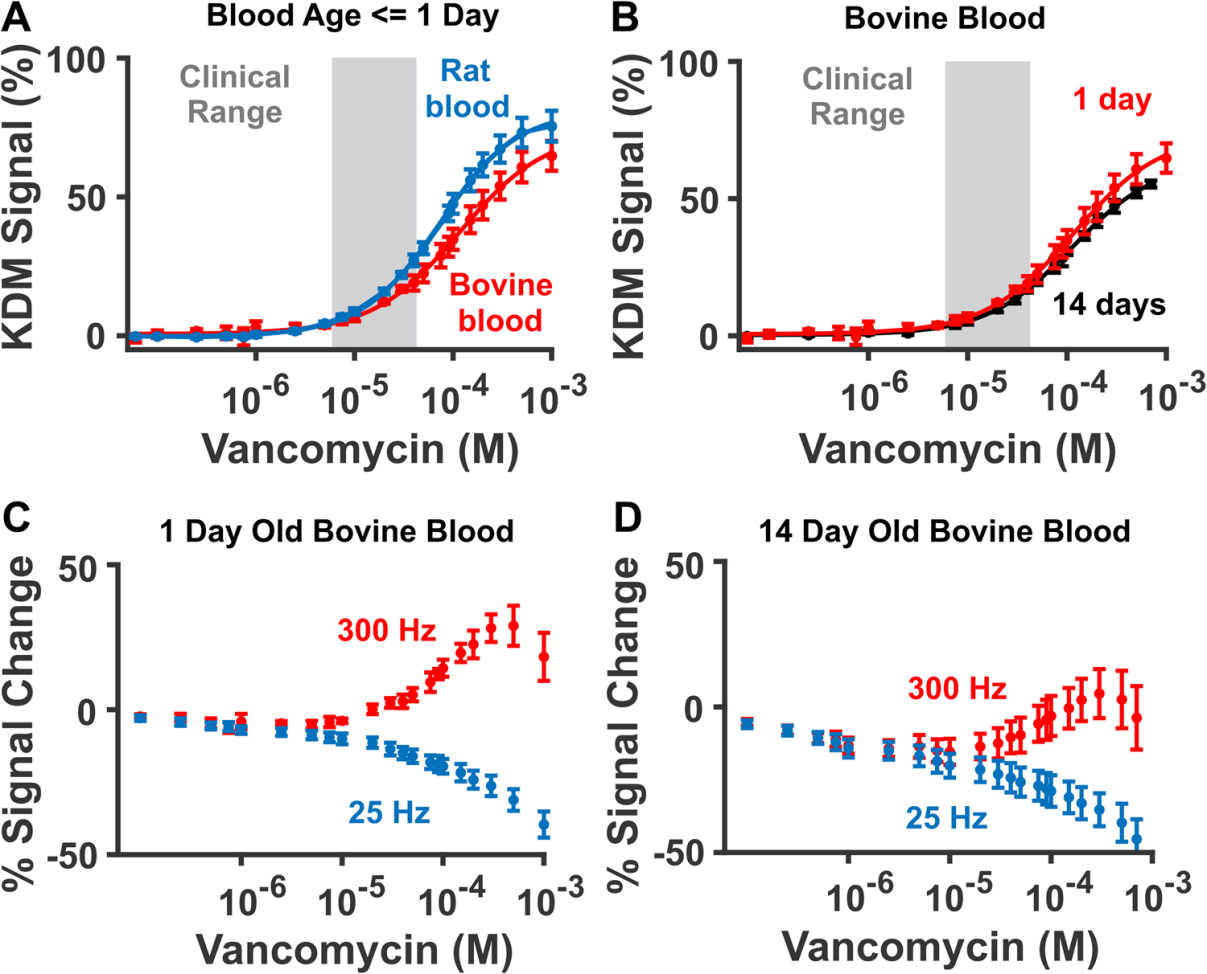
(**A**) Titrations performed in fresh rat blood and commercially sourced, one-day-old bovine blood produce distinct calibration curves. (**B**) To determine whether this is driven by species-specific differences in blood, or the greater age of the commercially sourced bovine blood, we compared calibration curves obtained in a bovine blood sample 1 day (red, K_1/2_ = 116 ± 15 µM) and 14 days (black, K_1/2_ = 112 ± 9 µM) after it was collected. Doing so, we find that the KDM response does not change significantly in the clinical range. (**C, D**) Examining the signal at the two square-wave frequencies used to perform KDM (here, 25 Hz and 300 Hz) we see that, for the 14-day-old blood, there is a notable signal decrease at vancomycin concentrations far below the aptamer’s dissociation constant. Given that no target-induced response should occur at these low concentrations, we believe this is an artifact due to time-dependent sensor degradation in the older blood.

Obtaining bovine blood from commercial vendors is often more convenient than collecting fresh blood samples each time one needs to generate a calibration curve. Unfortunately, however, calibration curves obtained in the commercially sourced and freshly-collected blood samples differ (Fig. 5A). Specifically, vancomycin sensors challenged in commercially sourced bovine blood yielded lower signal gain, which would lead to overestimated vancomycin concentrations (Fig. 5A). This difference in gain could arise from differences in the species source of the blood (bovine versus rat), how the blood was processed, or its age. Due to shipping, for example, commercially sourced blood is at least a day old by the time we use it. To determine whether blood age (time after collection) is an important calibration parameter, we titrated sensors in commercial bovine blood one day after collection and again using the blood from the same blood draw 13 days later. While the two samples yielded similar signals over the drug’s clinical range, the older sample produced lower signal at concentrations above this range (Fig. 5B). To assess the source of these differences, we examined the normalized square-wave signals at 25 and 300 Hz (Figs. 5C–D). Doing so, we find that while we observe a marked signal decrease at target concentrations below 1 µM (Fig. 5C–D), KDM effectively corrects for this. That there is still a difference in the 1 and 14 day old blood calibration curves suggests that blood age itself can impact the EAB sensor response. Given this, we believe measurements performed in the living body are best calibrated using curves obtained in the freshest possible blood.

**Figure 6:**
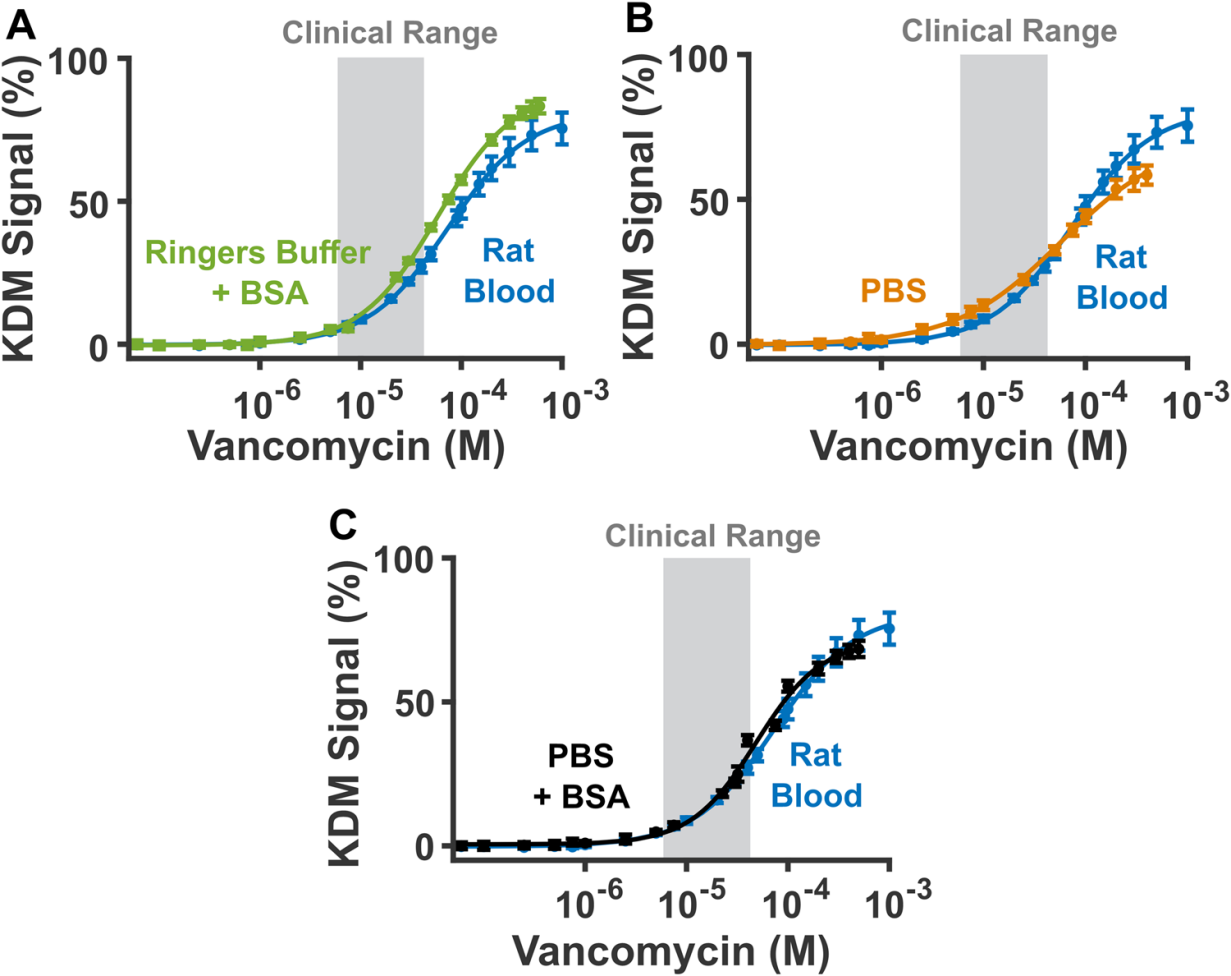
Here, we investigate whether simpler media can reproduce the response seen in freshly-collected rat blood. To do so, we compare titrations (KDM values derived from 25 and 300 Hz) collected in 37 °C freshly collected rat blood to (**A**) 37 °C Ringer’s buffer with 35 mg/mL bovine serum albumin (BSA), (**B**) 37 °C phosphate buffered saline (PBS) with 2 mM MgCl_2_, and (**C**) 37 °C PBS with 2 mM MgCl_2_ and 35 mg/mL bovine serum albumin. From these data we observe that calibration in 37˚C PBS with added BSA is a reasonable proxy for fresh rat blood. Of note, the close correspondence between the sensor’s response in simple buffers and in whole blood indicate that the sensor does not respond significantly to the components naturally present in blood.

Although it is the most accurate calibration matrix for in-vivo measurements, the collection of fresh blood for sensor calibration can be inconvenient, leading us to ask if there are other, more easily obtained media that can reproduce sensor behavior in that matrix. To investigate this, we produced calibration curves in Ringer’s buffer containing 35 mg/mL bovine serum albumin, phosphate buffered saline (PBS) containing 2 mM MgCl_2_, and PBS containing 2 mM MgCl_2_ and 35 mg/mL bovine serum albumin. We added bovine serum albumin to the buffered systems in an attempt to imitate the protein adsorption seen in blood^30^. In Ringer’s buffer (154 mM NaCl, 5.64 mM KCl, 2.16 mM CaCl_2_, 11.10 mM dextrose, 2.38 mM NaHCO_3_, 2 mM Trizma, and 35 mg/mL bovine serum albumin at pH 7.4), the titration yields greater signal gain than that observed in rat blood (Fig. 6A). Applying the resulting calibration curve, then, would lead to underestimation of vancomycin in rat blood. The PBS calibration curve, in contrast, exhibits higher response in the clinical range and a lower response above the clinical range. Thus, depending on the concentrations applied, this curve would lead to under- or overestimates of vancomycin in rat blood (Fig. 6B). A calibration curve collected in PBS with bovine serum albumin, however, reproduces the calibration curve obtained in fresh rat blood to a fair degree of accuracy (Fig. 6C). Enough so that, when we quantify the successive vancomycin additions in body temperature rat blood with this calibration curve, we obtain accuracies of better than 9% in the clinical range and better than 35% across all concentrations (Figure S8, Table S4). While poorer than the accuracy we achieve via calibration in rat blood, this calibration may still be acceptable in some applications. More broadly, these results suggest that buffered proxy systems can help guide development and application of sensors in vivo, and calibrate sensors when reduced accuracy is an acceptable tradeoff for improved convenience.

## Conclusions

With the appropriate selection of media temperature, composition, and age, a vancomycin-detecting EAB sensor easily achieves clinically relevant accuracy. Specifically, when calibrated at the appropriate temperature in freshly collected blood, sensors achieve accuracy of better than 10% when challenged under those same conditions. This result holds for both sensors calibrated against themselves and for sensors calibrated against other sensors. In contrast, calibration curves collected at inappropriate temperatures or in aged blood samples deviated more significantly from those seen in fresh, body temperature blood. Thus, these conditions may produce substantial over- or under-estimates of target concentration. Finally, in some cases, it is possible to use simpler media, such as phosphate buffered saline with bovine serum albumin, as a convenient and proxy for freshly collected blood for applications in which reduced accuracy is an acceptable trade-off relative to improved ease of calibration.

## Materials and methods

### Electrode fabrication

We fabricated sensors by soldering a 5 cm long, 75 µm diameter, PFA-coated gold wire (A-M Systems, Sequim, WA) to a gold-plated pin connector (CH Instruments, Inc., Austin, TX) with 60/40 lead-selenium solder (Digikey, Thief River Falls, MN). Before soldering, we used a digital caliper and razor blade to isolate 3 mm of gold on both ends of the gold wire. One end forms the working electrode, and the other end must be exposed for successful soldering. To prevent breakage of the delicate wires, we coated the wire-solder interface with a coat of urethane conformal coating (MG Chemicals, Surrey CA).

### Aptamer preparation

We first thawed a 2 μL, aliquot of 100 μM aptamer (sequence below) modified with a 6-carbon thiol linker on the 5′ end and methylene blue on the 3’ end (Biosearch Technologies, Novato, CA, dual HPLC purification, stored at − 20 °C).

5′-SH-(CH_2_)_6_- CGAGG GTACC GCAAT AGTAC TTATT GTTCG CCTAT TGTGG GTCGG-O-CH_2_-CHCH_2_OH-(CH_2_)_4_-NH-CO-(CH_2_)_2_-methylene blue-3′.

Because the manufacturer provides the DNA constructs in oxidized form, which does not effectively immobilize onto the gold surface, we must reduce the disulfide bond before deposition. To do so, we combined 2 μL of 10 mM Tris (2-carboxyethyl) phosphine (TCEP, Sigma-Aldrich, St. Louis, MO) with the aptamer sample for a 1 h thiol reduction in the dark at room temperature. We then diluted the sample with 96 μL 1X phosphate buffered saline (pH = 7.4). Here and elsewhere in our protocol, the 1X PBS is prepared by diluting 20X phosphate buffered saline (ChemCruz 20X phosphate buffered saline, Santa Cruz Biotechnology, Dallas, TX) to 1X using deionized water. , We quantified the concentration of the aptamer using the molar absorption coefficient at 260 nm provided by the supplier with UV–VIS spectroscopy (DU800, Beckman Coulter, Brea, CA). Using PBS, we then diluted the aptamer to 500 nM.

### Electrode preparation

For electrode cleaning and in-vitro characterization, our electrochemical cell contained a PFA-wrapped gold sensor working electrode, a platinum counter electrode (CH Instruments, Inc, Austin, TX), and an Ag|AgCl reference electrode (CH Instruments, Inc., Austin TX). We secured our working, counter, and reference electrodes in a cell vessel “shot glass” with a custom-fabricated Teflon lid fixture (See Fig. 1C). First, we electrochemically cleaned the electrodes in 0.5 M NaOH (Sigma-Aldrich, St. Louis, MO) by performing repeated cyclic voltammetry scans between − 1 and − 1.6 V (all potentials versus Ag|AgCl) at 1 V s^−1^ scan rate for 300 cycles (Table 1) using a CH Multipotentiostat (CHI1040C, CH Instruments, Inc.)^31^. We rinsed the electrodes in deionized water, then increased the surface area of the gold wire electrode by electrochemically roughening in 0.5 M H_2_SO_4_ (Sigma-Aldrich, St. Louis, MO) using a previously-described procedure that involves repeatedly stepping the potential between 0 and 2.2 V using chronoamperometry (Table 2)^31^. This electrochemical procedure, repeated 50 times using the CH instrument software, yields a 2 to fivefold increase in microscopic surface area^31^.

**Table 1:**
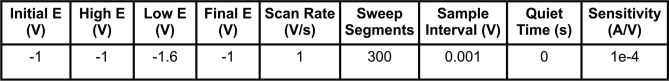
Cyclic Voltammetry parameters for electrochemically cleaning the electrodes in 0.5 M NaOH.

**Table 2:**
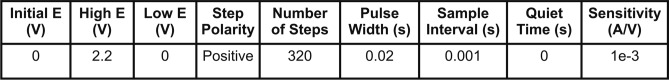
Chronoamperometry parameters for electrochemically roughening to electrodes in 0.5 M H_2_SO_4_.

Here, we repeat this procedure 50 times to increase the electrode surface area.

### Sensor fabrication

Immediately after roughening, we rinse the electrodes thoroughly in deionized water and place them in the prepared aptamer solution for 1 h at room temperature in dark conditions^8^. We then rinsed the electrodes with deionized water and immersed them for 12 to 18 h at room temperature in a 10 mM 6-mercapto-1-hexanol (Sigma-Aldrich, St. Louis, MO) in 1X PBS to passivate the surface. Following a final rinse with deionized water, the sensor was ready for use.

### Blood collection

12 mL of blood was collected from a single ~ 400 g adult male Sprague–Dawley Rat (Charles River Laboratory, Santa Cruz, CA). The rat was allowed to acclimatize to the colony for at least one week. The animal was placed under anesthesia using isoflurane gas and, following abolishment of toe pinch response, a horizontal incision was made below the rib cage. The incision was extended on each side to under the front legs, at which point vertical incisions were made through the ribcage. The diaphragm was then cut to reveal the heart and the septum pinned back using hemostats. A 20 mL syringe was prepared with 300 units of heparin in order to achieve a final concentration range of 20 units/mL (Sagent Pharmaceuticals, Schaumburg, IL). An 18G needle was attached to the top of the syringe and inserted into the left ventricle for blood collection (BD, Franklin Lakes, NJ). Very light pressure was placed upon the syringe in order to facilitate blood removal. The blood draw (15 mL total) was stopped when the flow of blood into the syringe finished after which point the animal was euthanized via isoflurane overdose. The Institutional Animal Care and Use Committee (IACUC) of the University of California at Santa Barbara approved our experimental protocol, which adhered to the guidelines provided by the American Veterinary Medical Association. Blood was transported immediately to a temperature-controlled bath at 37 °C, then used for experiments (Lauda Ecoline RE 106, Lauda-Brinkmann, Delran, NJ).

This study adhered to ARRIVE guidelines, where applicable. Specifically, randomization, blinding, and exclusion criteria (ARRIVE guidelines 3, 4, and 5) are not applicable because we drew blood from a single animal.

### Measurements

We filled the electrochemical cell with PBS, rinsed the electrodes in deionized water, and secured them in the electrochemical cell’s Teflon cap. Before each experiment, we collected three baseline cyclic voltammograms in PBS (− 0.1 to − 0.5 V versus Ag|AgCl, 0.1 V/s scan rate) to confirm successful aptamer deposition.

To perform a titration, we moved the electrode cap to a vessel of the selected media held in a temperature bath at the selected temperature (Lauda Ecoline RE 106, Lauda-Brinkmann, Delran, NJ). For bovine blood experiments, we commercially sourced blood from Hemostat Laboratories (Dixon, CA). For Ringer's buffer experiments, we prepared Ringer's buffer at pH 7.4 using 154 mM NaCl, 5.64 mM KCl, 2.16 mM CaCl2, 11.10 dextrose, 2.38 NaHCO3, 2 mM Trizma®, and 35 mg/mL bovine serum albumin (heat shock fraction, protease free, Sigma-Aldrich, St. Louis, MO). In the selected media, we collected square wave voltammetry scans (approximately − 0.2 to − 0.4 V versus Ag|AgCl, 25 mV amplitude) at 10, 25, and 300 Hz first in absence of target. We selected these frequencies because they were the originally reported signal-off, non-responsive, and signal-on frequencies for this particular aptamer. We observed, however, that at body temperature, 25 Hz yields a reliable signal-off response, and thus use this as the signal-off frequency for this work. Upon addition of incremental target concentrations, we back pipetted the electrochemical cell 15 times, allowed the solution to rest for two minutes, then proceeded with the measurement. To perform a spiking experiment, we used a CH software macro to repeatedly collect and store square wave voltammograms. We collected a 10 min baseline with no target, then sequentially added 10, 20, 50, 100, 300 µM vancomycin. For each addition, we pipetted rapidly, while taking care not to create bubbles in the blood, then measured the resulting signal for 5 to 10 min.

It is common to observe a downward drift in square wave voltammogram peak current, especially in complex media, such as whole blood. To correct for any peak signal loss, we applied a previously-described drift correction technique termed “Kinetic Differential Measurements” (KDM)^16^. Here, KDM denotes when normalized signal off-peak currents are subtracted from the normalized signal-on peak currents, then divided by the average of normalized signal-on and signal-off currents.

### Data analysis

To analyze square wave voltammetry peak currents in real time, we used a previously-reported, open source python script^32^. When provided with voltammogram bounds (typically, − 0.2 to − 0.4 V) and desired Savitzky–Golay filtering (5 mV), this script calculates peak height and KDM values. Raw data from this program was exported to Matlab for data analysis and presentation. To fit the data to a Hill-Langmuir isotherm, we input titration sconcentration and average KDM values into Matlab’s curve fitting application. We fit the data to the Eq. (1) with the following constraints: -infinity < KDM_max_ < infinity, 1e−12 < K_1/2_ < 1, -infinity < KDM_min_ < infinity, and 0 < n_H_ < 10. We present all fitting parameters in Table S3.

## Supplementary Information

Supplementary Information.

## Data Availability

The electrochemical datasets generated during and/or analyzed during the current study are available at figshare: 10.6084/m9.figshare.c.5907050.v1.
