# Supplementary material for: Improved calibration of electrochemical aptamer-based sensors
Source: Sci Rep. 2022 Apr 1;12:5535. doi: 10.1038/s41598-022-09070-7 (PMC8976050; doi:10.1038/s41598-022-09070-7)

Supporting Information

Improved Calibration of Electrochemical Aptamer-Based Sensors

Alex M. Downs^1,2^, Julian Gerson^3^, Kaylyn K. Leung^2,5^, Kevin M. Honeywell^3^, Tod Kippin^3,4^, and Kevin W. Plaxco^1,2,5*^

^1^Department of Mechanical Engineering, University of California Santa Barbara, Santa Barbara, CA 93106, USA.

^2^Center for Bioengineering, University of California Santa Barbara, Santa Barbara, CA 93106, USA.

^3^Department of Psychological and Brain Sciences, University of California Santa Barbara, Santa Barbara, CA 93106, USA.

^4^The Neuroscience Research Institute and Department of Molecular Cellular and Developmental Biology, University of California Santa Barbara, Santa Barbara, California 93106, United States

^5^Department of Chemistry and Biochemistry, University of California Santa Barbara, Santa Barbara, CA 93106, USA.

*Corresponding author: [kwp@ucsb.edu](mailto:kwp@ucsb.edu)

**Table S1:** Employing “out of dataset” calibration curves does not significantly reduce accuracy over the clinically relevant target concentration range. Here, we challenged four sensors, in each case using the average calibration curve of the other three sensors.


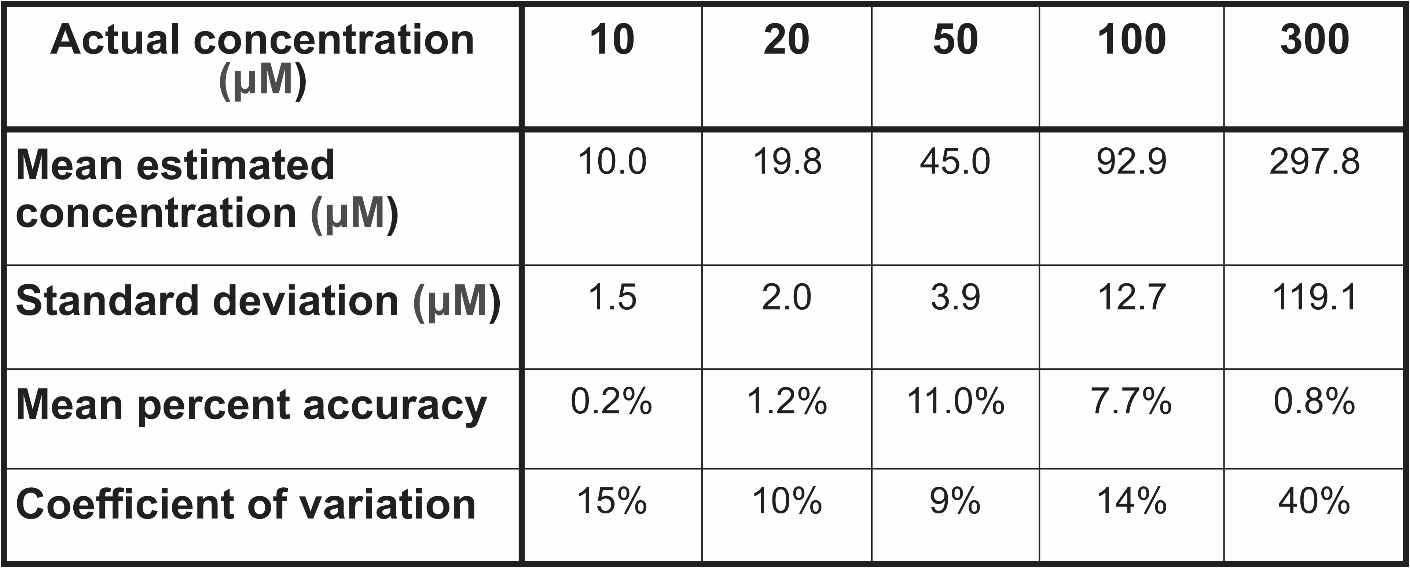


**Table S2:** Calibrating each individual sensor does not significantly improve accuracy over the clinically relevant target concentration range. To show this, we used the Hill-Langmuir isotherm calibration curve of each individual electrode to produce concentration estimates using that same electrode when challenged with a series of vancomycin additions in 37°C fresh rat blood.


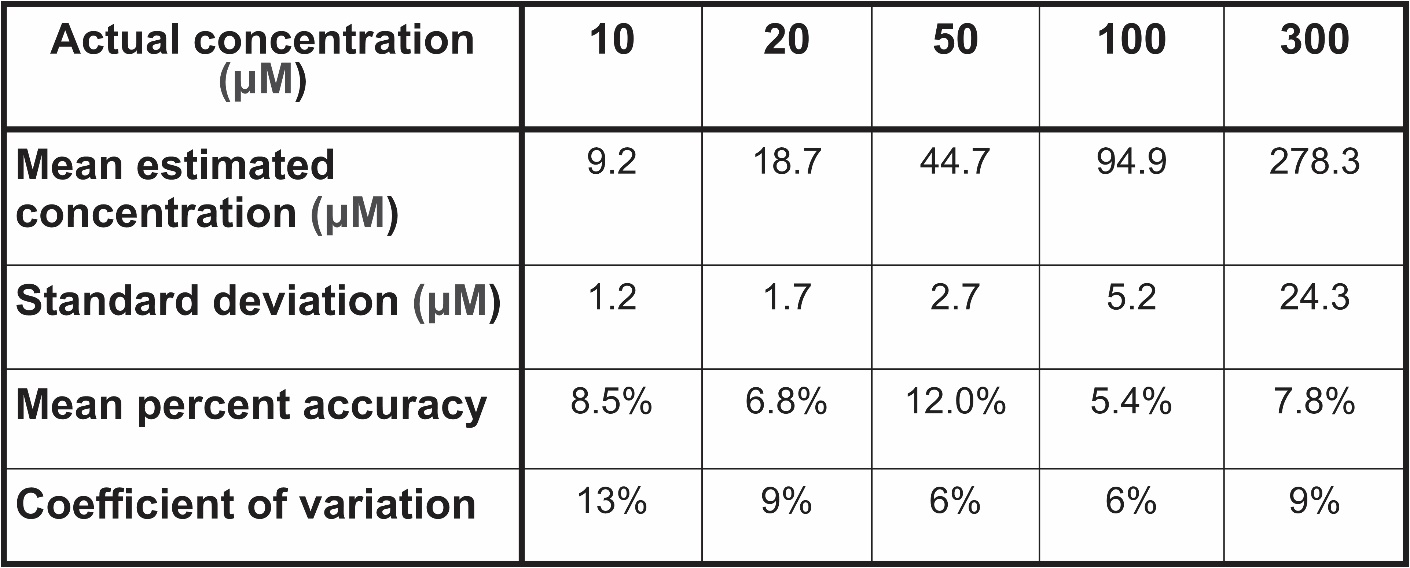


**Table S3:** Hill-Langmuir parameters for the media conditions presented (rat blood, bovine blood, Ringer’s buffer with bovine serum albumin, phosphate buffered saline, and phosphate buffered saline with bovine serum albumin,


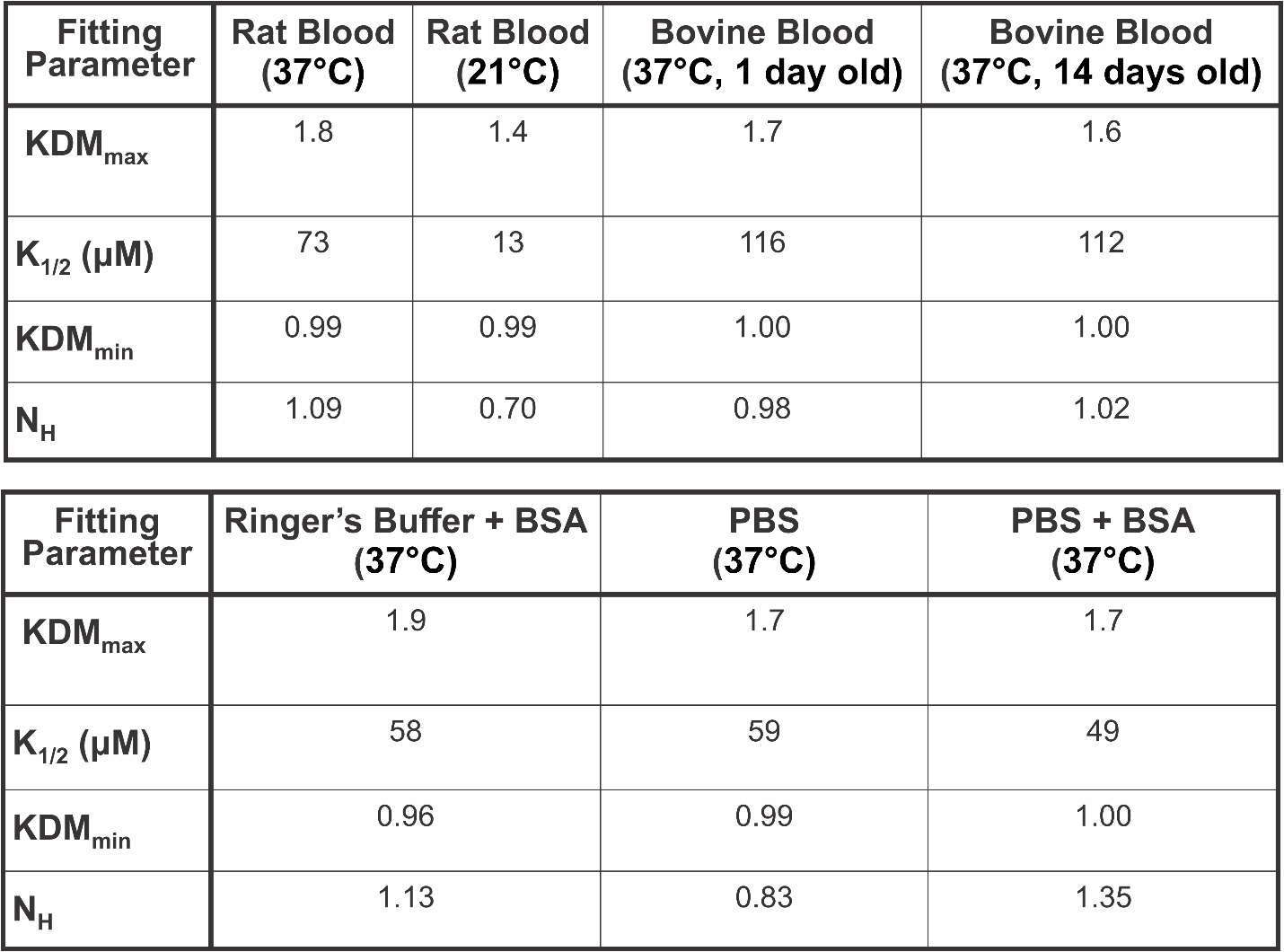


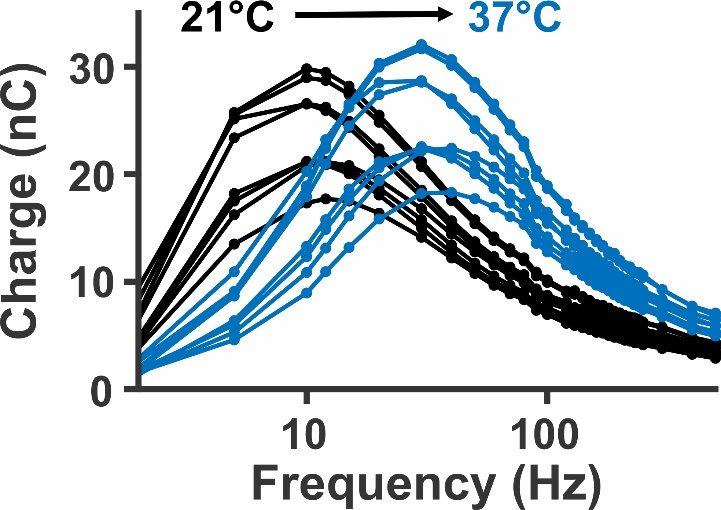


**Figure S1:** Between room temperature (black) and body temperature (blue), peak charge transfer shifts toward higher frequencies, indicating an increase in electron transfer rate. Shown are plots of charge transfer versus square-wave frequency for eight sensors. Note that variation in ethe total charge transferred occurs due to differences in electrode gold surface area, which changes the number of methylene blue redox reporters for a given sensor.


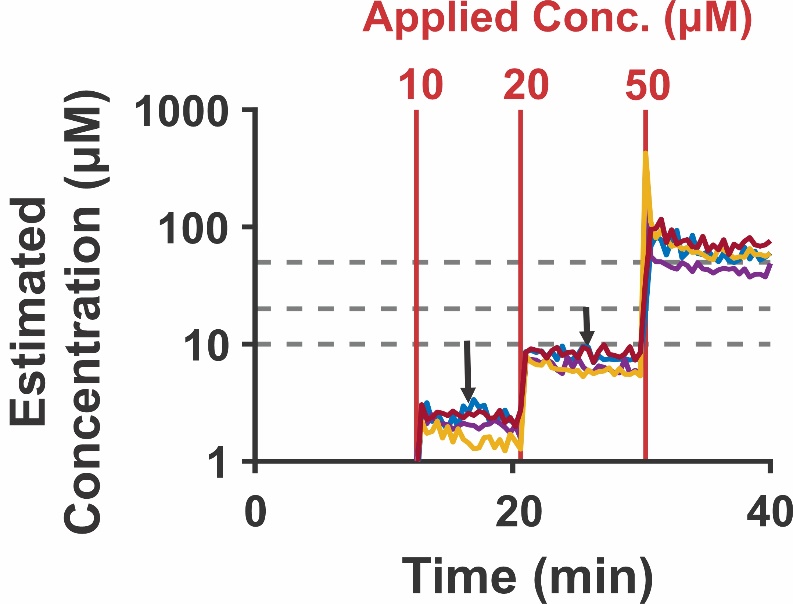


**Figure S2:** Calibrating EAB sensor data collected at body temperature with a calibration curve collected at room temperature fails produces substantial under-estimates of vancomycin concentration at 10 and 20 µM doses. Doses at 100 and 300 µM are not shown because, due to differences in the observed KDM values, the room temperature calibration curve fail to quantify at these concentrations.


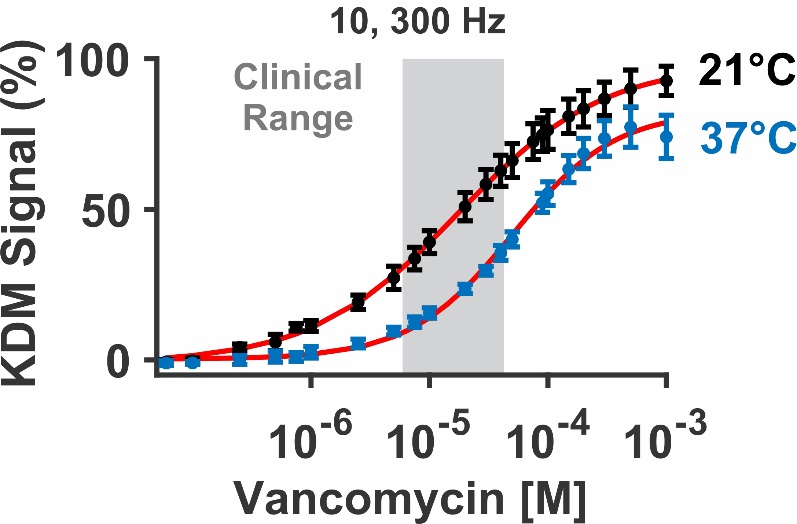


**Figure S3:** EAB sensor response is sufficiently temperature dependent that the change in signaling between room and body temperature is substantial. Here, we compare titrations in freshly collected rat blood at 21°C and 37°C. Calibration curves collected at 10 and 300 Hz at 21°C (black, n = 4 sensors, K_1/2_ = 175 ± 1 µM) and 37°C (blue, n = 4 sensors, K_1/2_ = 499 ± 8 µM) yield a 20 to 25 % difference in clinical range response magnitudes.


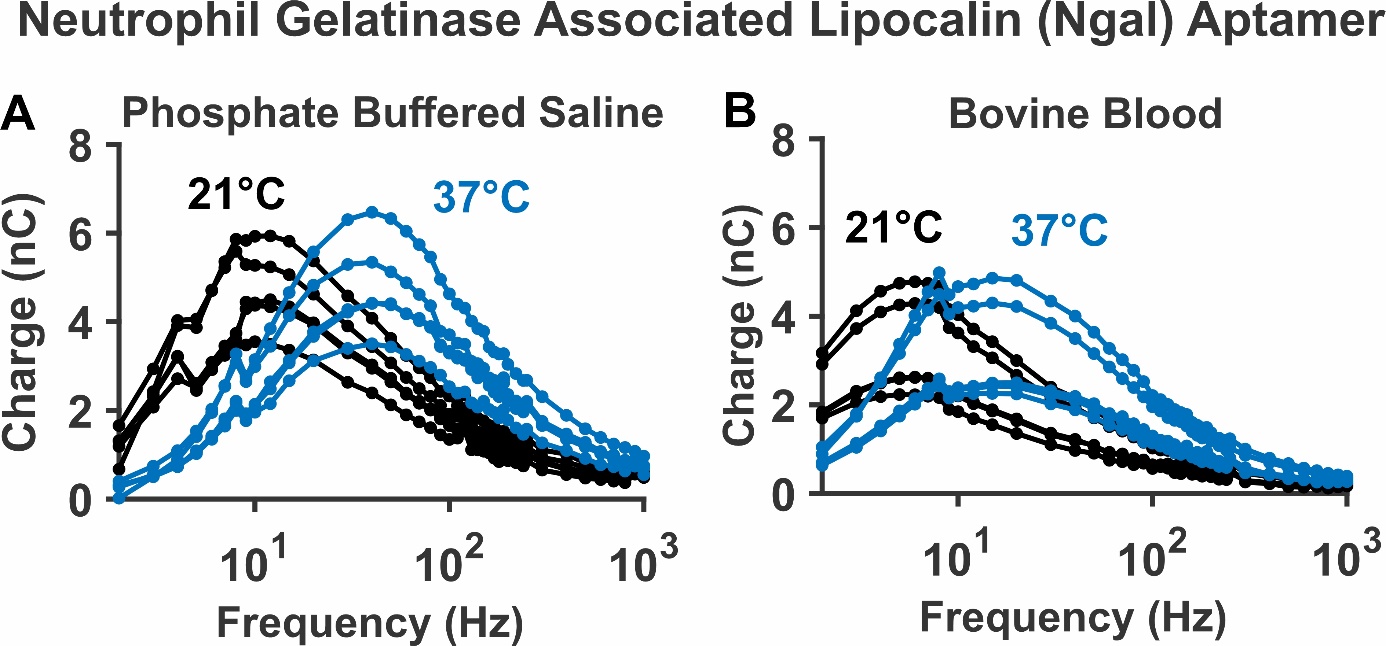


**Figure S4:** For an aptamer responding to the protein target, Ngal, electron transfer changes substantially between room (20˚C) and body (37˚C) temperature. **(A)** In phosphate buffered saline, the peak charge transfer shifts by 30 Hz (from a frequency of approximately 10 Hz to 40 Hz). **(B)** In whole bovine blood, the peak location shifts by 10 Hz (from approximately 5 Hz to 15 Hz).


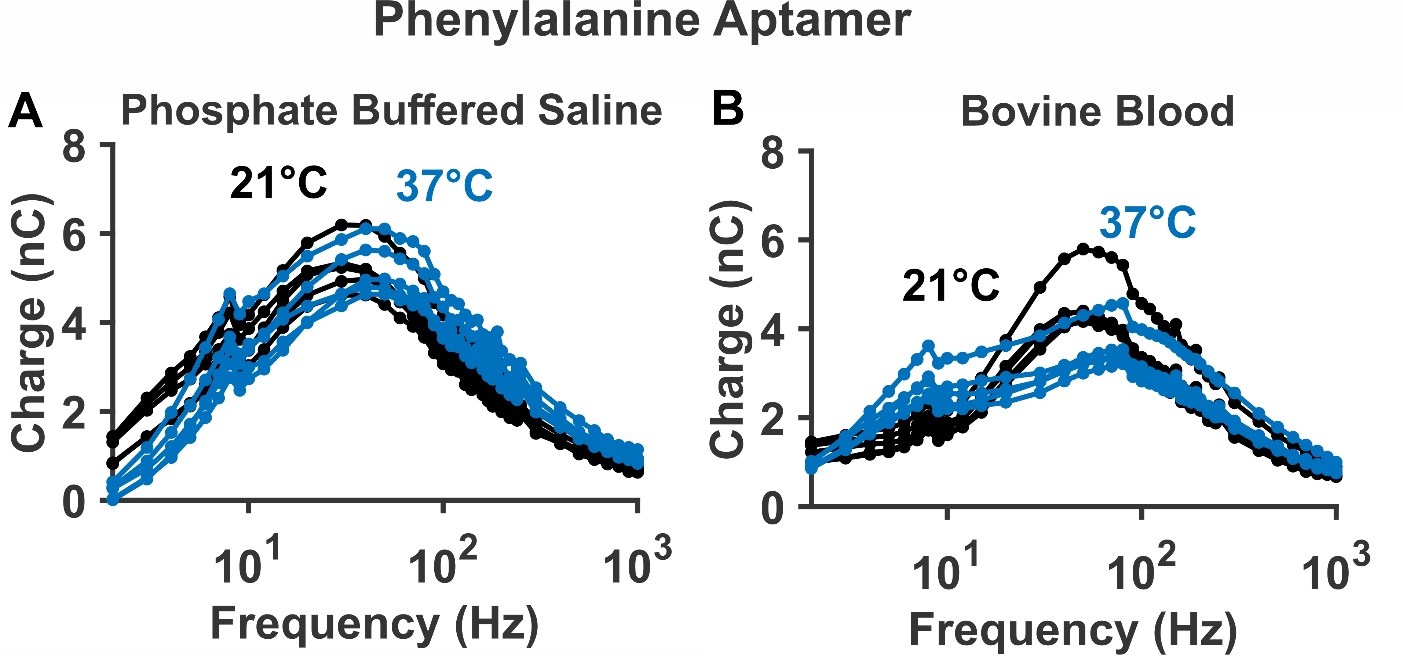


**Figure S5:** For an aptamer responding to the amino acid target, phenylalanine, electron transfer changes slightly between room (20˚C) and body (37˚C) temperature. **(A)** In phosphate buffered saline, the peak charge transfer shifts by approximately 10 Hz (from 30-40 Hz to 40-50 Hz). **(B)** In whole bovine blood, the peak broadens, and a slight secondary peak appears at frequencies near 10 Hz.


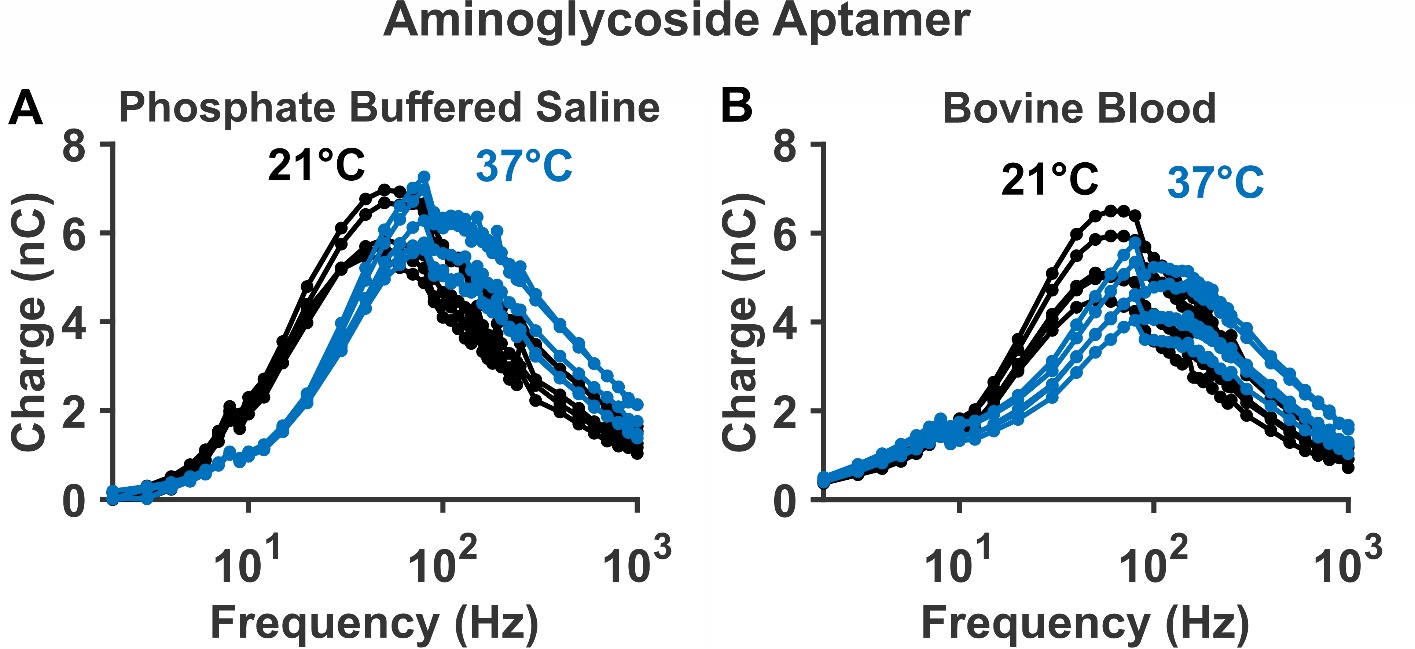


**Figure S6:** For an aptamer responding to aminoglycoside antibiotics, electron transfer changes substantially between room (20˚C) and body (37˚C) temperature. This occurs in both **(A)** phosphate buffered saline and **(B)** undiluted bovine blood.


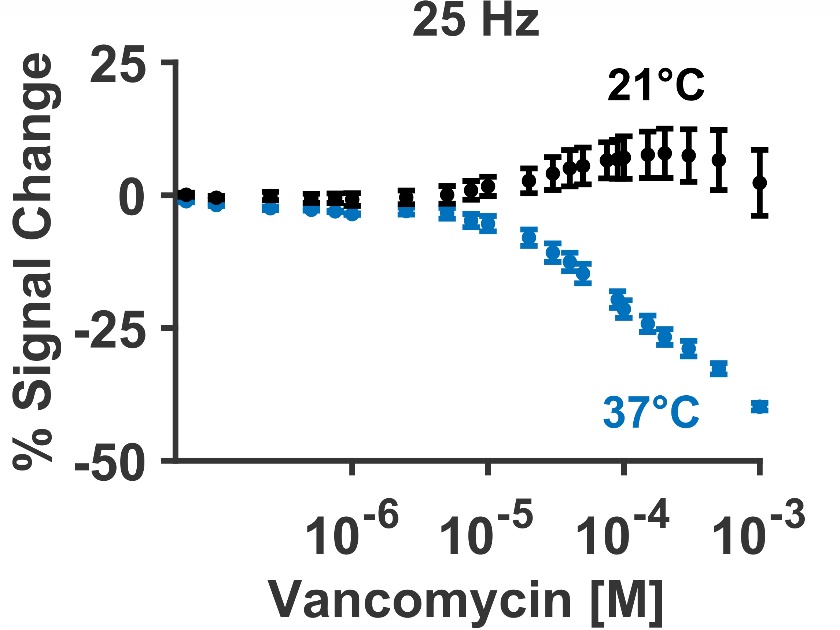


**Figure S7:** Between room temperature (black) and body temperature (blue) the normalized peak signal observed at 25 Hz changes from a weak signal-on response to a clear signal-off response.


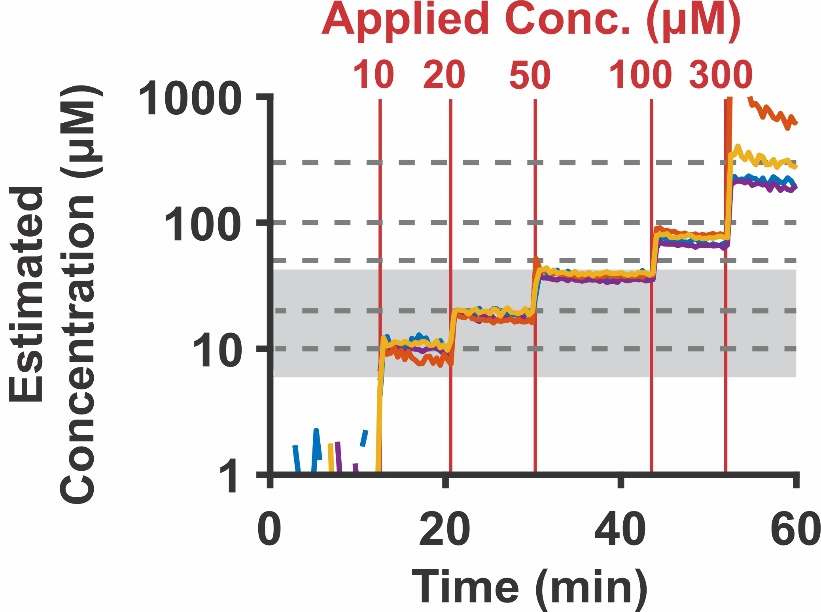
­

**Figure S8:** We calibrate the response of our sensors in body temperature rat blood using a titration curve collected in body temperature PBS with 2 mM MgCl_2_ and 30 mg/mL BSA (dotted lines indicating 10, 20, 50, 100, and 300 µM vancomycin, grey area indicated clinical range)

**Table S4:** Calibrating each data collected in body temperature rat blood with a calibration curve collected in body temperature PBS with 2 mM MgCl_2_ and 30 mg/mL BSA produces measurement accuracy of greater than 35%.


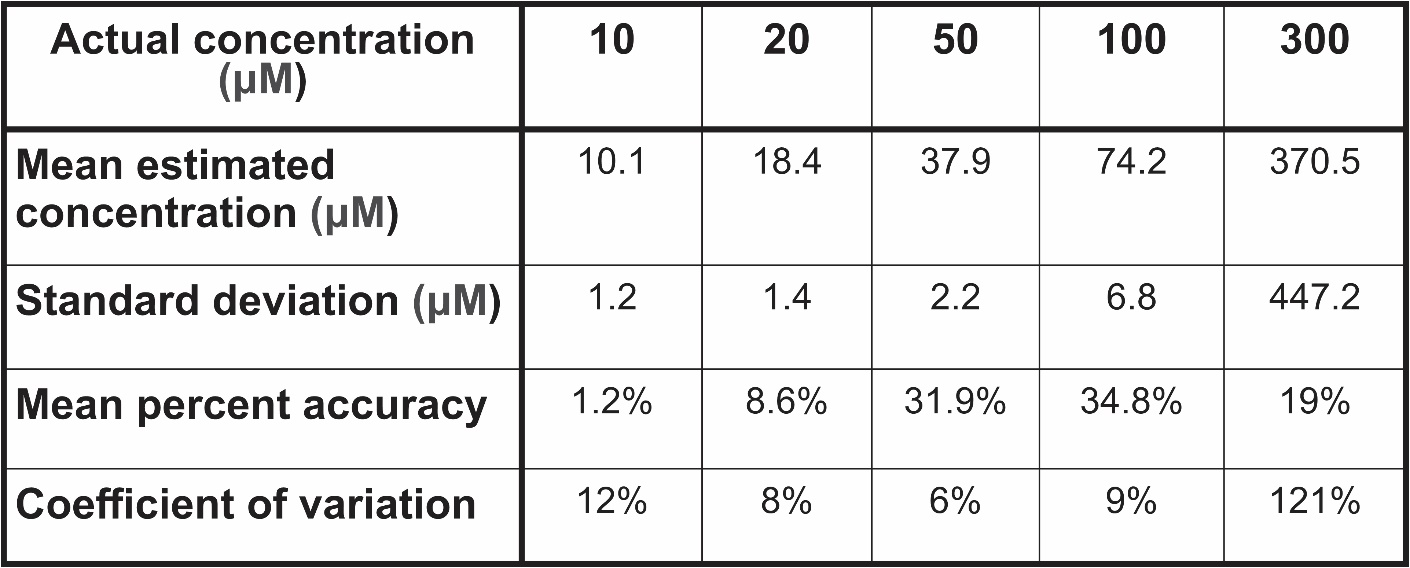

Supplement: Supplementary file 1 — Supplementary Information. [file 41598_2022_9070_MOESM1_ESM.docx]
